# Molecular detection, not extended culture incubation, contributes to diagnosis of fungal infection

**DOI:** 10.1186/s12879-021-06838-6

**Published:** 2021-11-15

**Authors:** Alex Zhu, Teresa Zembower, Chao Qi

**Affiliations:** 1 Northwestern University, IL Evanston, USA; 2 Department of Pathology, Clinical Microbiology Laboratory, Northwestern University Feinberg School of Medicine, Northwestern Memorial Hospital, 303 E. Chicago Ave., Chicago, IL 60611 USA; 3 Department of Medicine, Division of Infectious Diseases, Northwestern University Feinberg School of Medicine, Chicago, IL USA

**Keywords:** Fungal infections, Fungal culture, ITS PCR

## Abstract

**Background:**

Despite its low sensitivity, fungal culture remains one of the key methods for diagnosis and treatment of fungal infections, as it identifies the etiology at the genus and species level and affords the opportunity for susceptibility testing. The *Manual of Clinical Microbiology* recommends that fungal culture screening for all pathogens should routinely be held for 4 weeks to maximize the recovery of slow-growing species. Information on the optimal fungal culture time in this era of expansion of immunocompromised populations and availability of molecular diagnostics is lacking. We reviewed our experience with fungal culture to determine the optimal culture incubation time. In addition, our experience of broad-range ITS PCR for diagnosis of culture-negative fungal infections was also reviewed.

**Methods:**

Fungal culture and ITS PCR results from January 1, 2013, to December 31, 2017, were reviewed.

**Results:**

This study included 4234 non-duplicated positive cultures. *N*inety-six percent (4058) of the positive cultures were detected in the first 7 days of incubation. During the second week of incubation, 111 (2.8%) positives were detected from day 8 to day 10, and 71 (1.7%) were detected from day 11 to day 14. Only 6 (0.1%) positive cultures were detected in the third week of incubation, and no positive culture was detected in the fourth week of incubation. No clinically significant fungal isolates were recovered after 14 days. Clinically significant pathogens were detected in 16 (0.2%) culture-negative samples by ITS PCR.

**Conclusion:**

Extending culture incubation beyond 2 weeks did not generate clinically relevant results. When culture failed to make a laboratory diagnosis, broad-range internal transcribed spacer (ITS) rRNA gene PCR followed by sequencing produced clinically significant results.

## Background

As the population of immunocompromised patients continues to expand as a result of widespread adoption of aggressive immunosuppressive therapies and use of new immune-modifying drugs, invasive fungal infections are increasing in both number and severity [1–3]. The 12-week mortality rate of patients with invasive fungal infections was reported to be as high as 46.7% for adult hematopoietic stem cell transplant recipients and 29.6% for solid organ transplantation patients [4, 5]. Prompt initiation of antifungal therapy is the most important intervention to decrease mortality in patients with invasive fungal infection [6].

Despite its low sensitivity, fungal culture remains one of the key methods for diagnosis and treatment of fungal infections, as it identifies the etiology at the genus and species level and affords the opportunity for susceptibility testing. Recovery of fungal pathogens relies on a number of factors, including specimen quality, appropriate specimen collection and transport, and optimal incubation condition and time. Incubation of fungal culture for 4 weeks is a universal recommendation based on empirical experience [7]. The *Manual of Clinical Microbiology* endorses this practice and recommends that fungal culture screening for all pathogens be routinely held for 4 weeks to maximize the recovery of slow growing species. A few studies have investigated the fungal culture incubation time and generated mixed results. Morris et al. reviewed over 2700 consecutive clinical cultures and determined that 98% of fungal isolates were detected by day 14 [8]. Labarca et al. evaluated close to 4000 positive fungal cultures and concluded that isolates recovered during the fourth week were rarely clinically significant [9]. Bosshard’s study suggested that 2 weeks of incubation was sufficient for evaluation of non-dermatophytes, whereas a 4-week incubation was necessary for recovery of dermatophytes [10]. Hove and Woods reviewed their experience in an area endemic for *Histoplasma capsulatum* and demonstrated that 18 (29%) of 62 *H. capsulatum* isolates were recovered in weeks 3 and 4 [11].

To overcome the poor sensitivity of fungal culture, additional diagnostic tests have been developed. Tests for antigen and fungal biomarkers are often used in addition to culture to help establish a diagnosis [12]. PCR-based assays targeting various genetic regions have been developed to improve the diagnostic yield but are mostly restricted to *Aspergillus* and *Candida* species [13]. In situations when other opportunistic pathogens cannot be ruled out, molecular detection of a broad range of pathogens is desired. Broad-range ITS rRNA gene PCR followed by sequencing holds promise for detection and identification of fungal pathogens in human samples [12]. In a prospectively performed study, ITS PCR demonstrated its value for diagnosis of microscopy-negative fungal infections [14].

ITS PCR was implemented in our lab in 2013. Our protocol requires infectious disease physicians to specially request the test when a specimen from a sterile source is fungal culture-negative and the suspension of fungal infections is high. The purpose of this study was to review our experience with fungal culture to determine the optimal culture incubation time, and to review our experience of ITS PCR for diagnosis of culture-negative fungal infections.

## Methods

### Study design

We retrospectively reviewed the positive fungal cultures of clinically significant specimens recovered in the Clinical Microbiology Laboratory at Northwestern Memorial Hospital (NMH), a 900 bed-teaching hospital in Chicago, between January 1, 2013, and December 31, 2017. Specimen types included abscesses, body fluids, bronchoalveolar lavage, endotracheal aspirate, sputum, skin, tissues, and wound. Information collected included fungal smear result, culture date, growth detection date, and species identification. Time to culture positivity was counted in days from when the culture was started to the time when fungal growth was first detected.

### Fungal stain and culture

Microscopic analysis of the fungal smear was performed on all specimens with a wet mount in 20% KOH and with Calcofluor-white staining. The smear of sterile body fluids was prepared with cytospin centrifugation. Clinical specimens were cultured on inhibitory mold agar plates and brain and heart infusion agar plates. Plates were incubated at 30 °C in ambient air for 4 weeks. All plates were examined 3 times a week during the 2 weeks of incubation and then once a week thereafter. The time to detection of fungal growth was counted in days from when the culture was started to the time when fungal growth was first detected. Identification was based on macroscopic and microscopic morphologies of culture using the “Medically Important Fungi—A Guide to Identification 5th Edition” as a reference.

### ITS PCR

During the study period, infectious disease physicians had access to broad-range ITS PCR for culture-negative patients with high clinical suspicion of fungal infections. The test was offered as an add-on test performed using the residual sample after the specimen was cultured. Only specimens from sources free from colonization by fungal organisms were tested. When ITS PCR became positive, identification was determined with sequencing, and the identification result was discussed with the treating physician to obtain clinical correlation before final reporting.

DNA extraction for ITS PCR was performed in the clinical microbiology laboratory at Northwestern Memorial Hospital using established protocols. Briefly, 200 µL of fluid or 0.03 grams of solid were used for nucleic acid extraction. DNA was extracted with the QIAamp DNA Mini Kit (Qiagen Sciences, LLC, Louisville, KY). PCR amplification of the ITS region was performed with primers ITS1 5’- TCCGTA GGTGAACCTGCG G- 3’ and ITS4 5’- TCCTCCGCTTATGATATG C - 3’ as described preciously [15]. Each PCR reaction (50 µL) consisted of 10x Buffer, Taq polymerase (Invitrogen Platinum taq DNA polymerase high fidelity), 50 mM MgSO¬4, dNTP, DNase/RNase-free H20, and 4 µL DNA template. To ensure the quality of the nucleic acid extraction and PCR process, 3 sets of controls were included with each PCR run. An internal extraction/inhibition control using primers to the Beta globin gene was included to account for false-negative results. Sterile water and *Candida albicans* ATCC® 10,231 were used as negative and positive DNA controls, respectively. Patient samples and controls were run on a C1000 Touch Thermal Cycler (Bio-Rad Laboratories, Hercules, CA) with the following conditions: denaturation 95 °C for 5 min, 34 cycles of denaturation for 30 s, annealing at 56 °C for 30 s, and extension at 68 °C for 1 min. Following amplification, gel electrophoresis was performed to determine whether fungal nucleic acid was present in the specimen and whether the controls performed as expected. The PCR product was purified using the Qiagen QIAquick PCR Purification Kit (Qiagen, Valencia, CA) prior to sequencing. Sequencing was performed on the ABI 3500 Sequencer (Thermal Fisher Scientific, Waltham, MA) on the eluted DNA. Identification was obtained by BALST search against the National Center for Biotechnology Information’s GenBank (https://blast.ncbi.nlm.nih.gov/Blast.cgi). An identification score of ≥99% was used to determine the identification. To be consistent with the report of culture results, species identification was reported for yeast isolates and dimorphic fungi; genus identification was reported for other fungal species unless species identification was requested by clinicians. The assay was developed by the NMH clinical microbiology laboratory. Its performance was assessed by testing 125 clinical samples and compared to the culture results. Using the same identification reporting scheme for culture, the sensitivity and specific were 100% and 98.4% respectively. Identification generated for culture negative samples was confirmed by single target qPCR. Limits of detection were determined by detection of *Candida albicans* (ATCC 10231) in the positive control using a series dilution across a 5-log range (10, 10^2^, 10^3^, 10^4^, 10^5^). The actual colony count in the suspension was determined by plating the sample on Sabrau Dextrose Agar for 7 days. The limit of detection of the assay was approximately 1000 CFU/mL.

### Medical record review

Electronic medical records were reviewed to determine the clinical significance of the organisms recovered by culture after 14 days of incubation and the organisms detected by ITS PCR. A result was determined to be clinically significant when the treating physician’s diagnosis based on the test result was recorded in the patient chart and the patient was treated for the identified organism.

## Results

### Time to growth detection

This study included 4234 non-duplicated positive cultures. Table 1 lists the fungal species recovered by culture. Cultured specimens include abscess, blood, eye (corneal scraping, vitreous fluid), skin, and respiratory tract samples (sputum, endotracheal aspirate, BAL fluid), sterile body fluids (CSF, pericardial, peritoneal, and synovial fluids), and tissues. A positive fungal smear was detected in 1219 (28%) samples. Of the 4234 positive cultures, 4058 (95.8%) were detected by day 7 of incubation, 111 (2.8%) were detected from days 8 to 10; 71 (1.7%) from days 11 to 14 and only 6 (0.1%) from days 15-21. No positive culture was detected in the fourth week of incubation. (Table 1).

**Table 1:** Time to detection of fungal growth

Species*	Number (% of total)	Smear positive (%)	Days to positive <=7	Days to positive 8–10	Days to positive 11–14	Days to positive 15–21	Days to positive 22–28
*Candida*	3493 (82.6 )	1009 (28.9)	3468 (99.3)	23 (0.6)	2 (0.1)	0 (0)	0
*Aspergillus*	244 (5.8)	94 (38.5)	231 (94.7)	13 (5.3)	0 (0)	0 (0)	0
*Penicillium*	117 (2.8)	2 (1.7)	85 (72.6)	14 (12)	15 (12.8)	3 (2.6)	0
Dimorphic fungi^a^	99 (2.3)	37 (37.4)	40 (40.4)	22 (22.2)	37 (37.4)	0 (0)	0
Other yeasts^b^	59 (1.4)	20 (33.9)	58 (98.3)	1 (1.7)	0 (0)	0 (0)	0
Melanized fungi^c^	67 (1.6)	10 (14.9)	36 (53.7)	20 (29.9)	11 (16.4)	0 (0)	0
Other hyaline Fungi^d^	44 (1.0)	7 (15.9)	38 (86.4)	2 (4.5)	1 (2.3)	3 (6.8)	0
*Scedosporium*	36 (0.9)	16 (44.4)	28 (77.8)	7 (19.4)	1 (2.8)	0 (0)	0
Mucorales^e^	34 (0.8)	15 (44.1)	34 (100)	0 (0)	0 (0)	0 (0)	0
Dermatophytes^f^	22 (0.5)	3 (13.6)	10 (45.4)	8 (36.4)	4 (18.2)	0 (0)	0
*Crytpococcus neoformans*	16 (0.3)	6 (37.5)	15 (93.8)	1 (6.2)	0 (0)	0 (0)	0
Total	4234	1219 (28.8)	4058 (95.8)	111 (2.6)	71 (1.7)	6 (0.1)	0

*Identification was based on macroscopic and microscopic morphologies of culture using the “Medically Important Fungi—A Guide to Identification 5th Edition” as a reference; .^a^Diamorphic fungi include *Blastomyces dermatitidis*, *Coccidioides immitis*, *Histoplasma capsulatum*, *Sporothrix schenckii*; ^b^Other yeasts include *Saprochaete*, *Rhodotorula*, *Saccharomyces cerevisiae, Trichosporon*; ^c^Melanized fungi include *Alternaria*, *Aureobasidium*, *Cladosporium*, *Curvularia*, *Epicoccum*, *Exophiala*, *Fonsecaea*, *Ochroconis*, Phialophora; ^d^Other hyaline fungi include *Acremonium*, *Beuveria*, *Fusarium*, *Paecilomyces*, *Scopulariopsis*, *Trichoderma*; ^e^Mucorales include *Cunninghamella*, *Mucor*, *Rhizopus*, *Rhizomucor*, *Syncephalastrum*; ^f^Dermatophytes include *Trichophyton*, *Microsporum*

The time required for recovery of different species varied. Of 4058 (95.8%) cultures growing yeast, including *Candida* specie*s*, *Cryptococcus neoformans*, and other yeast species, all but 2, were recorded as positive after fewer than 10 days of incubation. Growth of *Candida* was detected in 2 cultures on day 11. Growth of *Aspergillus* species and Mucorales was not detected after day 10. Fungal species belonging to dimorphic fungi, melanized fungi, and dermatophytes required a longer incubation time, with growth of 59.6% dimorphic fungi, 46.3% melanized fungi, and 52.2% dermatophytes detected in the second week of incubation. The time to detection of different fungal groups is listed in Table 1.

### Clinical significance of fungal isolates recovered after 14 days

The patients with a positive culture detected after 14 days were further investigated to determine the clinical significance of the recovered organisms. Three patients (patient 1, 2, 5) had no sign of infection at the specimen collection site and did not receive treatment for the recovered organism. The symptoms of the other 3 patients (patient 3, 4, 7) were attributed to either bacterial or viral infections based on the clinical course and treatment response. The recovered fungal organisms had no clinical significance. The last patient in the group (patient 6) was evaluated for a lung mass. Bronchial wash was collected. The patient was later diagnosed with squamous cell lung carcinoma. *Penicillium* species was recovered from the bronchial wash had no clinical significance. A summary of patient history and clinical information is provided in Table 2.

**Table 2:** Patients with positive culture after 14 days of incubation

Patient	Specimen	Culture identification	Time to detection of growth (days)	Patient history	Reason for culture	Clinical information
1	Skin Biopsy	*Paecilomyces* spp.	19	Renal transplantation	One month of scattered papular lesions on extremities	No finding to suggest for infection by surgical pathological examination.
2	BAL	*Acremonium* spp.	20	Lung transplant	Left ureteral stone	No findings to suggest for infection by chest X–ray.
3	Sputum	*Acremonium* spp.	22	HIV on HAART	Suspected for hospital acquired pneumonia	Patient responded well to vancomycin and pipera cillin/tazobactam treatment.
4	Sputum	*Acremonium* spp.	25	Bioprosthetic aortic valve	Hemoptysis with abnormal chest X ray	Hemoptysis resolved and serial CXRs showed resolution of persistent RLL infiltrate after empiric antibiotic treatment.
5	Wound	*Penicilium* spp.	15	Colon carcinoma and asthma	Evaluation prior to planned ventral hernia repair surgery	No infection
6	Bronchial wash	*Penicilium* spp.	15	No significant past medical history	Lung mass	Sequamous cell carcinoma
7	BAL	*Penicilium* spp.	19	No significant past medical history	Cough, fatigue & decreased appetite	Upper respiratory viral infection

### Broad-range ITS PCR for diagnosis of fungal infections

Five hundred twenty-three samples were tested with ITS PCR. Clinically significant pathogens were detected in culture-negative samples from 16 (0.3%) patients, including 5 patients with acute myeloid leukemia, 6 patients who underwent transplantation, 2 patients with no significant past medical history, 1 cancer patient, 1 pregnant patient, and 1 patient with a ventriculoperitoneal shunt for hydrocephalus (Table 3). Presence of a morphologically consistent fungal organism was observed on the fungal smear in 9 (56%) samples. The identifications were evaluated by the clinical team, and the patients received treatment for the identified organisms.

**Table 3:** Identification by ITS sequencing

Patient history	Clinical indication for fungal culture	Specimen	Direct microscopic examination	Identification by ITS sequencing
Acute myeloid leukemia (AML)	Disseminated fungal infection involving multiple organs	Apical mass, liver biopsy, pericardium	Positive	*Saccaromyces cerevisiae*
Recent history of pneumonia requiring hospitalization	Left cerebellar lesion on imaging	Brain tissue	Positive	*Blastomyces dermatitidis*
Heart transplantation	Cavitary pneumonia	BAL	Negative	*Rhizopus microsporus*
Renal transplantation	Multiloculted cystic lesion medial to transplanted kidney in left pelvis	Blood	Negative	*Histoplasma capsulatum*
Hydrocephalus with a ventriculoperitoneal shunt	Brain lesion	Brain tissue	Positive	*Rhodotorula* spp.
Renal transplantation	Cavitary lung lesions/pulmonary nodule	CSF	Negative	*Aspergillus*
AML	Pneumonia, sinusitis and periorbital cellulitis	Ethmoid	Negative	*Fusarium* spp.
Stage III Wilms tumor	Skin lesion	Scalp lesion	Positive	*Rhizopus* spp.
AML	Cutaneous fungal lesions	Skin biopsy	Positive	*Fusarium* spp.
Pregnancy	Severe amniotic fluid infection	Placenta tissue	Positive	*Candida glabrata*
AML	Neutropenic fevers and pulmonary nodules	Tissue	Negative	*Aspergillus* spp.
AML	Disseminated fungal infection with empyema, pericarditis	Pericardial abscess	Positive	*Candida tropicalis*
Renal transplantation	Pulmonary cavitary lesion	TISC-left upper lobe region	Negative	*Cunningmahella* spp.
No significant medical history	Liver abscess	Liver biopsy	Positive	*Candida lusitaniae*
Renal transplantation	Disseminated invasive Aspergillosis involving multiple organs	Brain tissue	Positive	*Aspergillus* spp.
Renal transplantation	Mass in the left sinus invading the orbit	Eye tissue	Negative	*Aspergillus* spp.

## Discussion

Our experience with fungal culture and ITS PCR from 2013 to 2017 was reviewed in this study. Four thousand, two hundred thirty-four non-duplicated positive cultures from clinically significant sources were reviewed for time to positivity. Even though the time required for recovery of different species varied, growth was detected in fewer than 14 days in 99.9% of cultures. Organisms recovered after 14 days had no clinical significance. ITS PCR yielded clinically significant positive results for culture-negative samples from patients with high suspicion of fungal infection.

The *Manual of Clinical Microbiology* recommends that cultures that screen for *Candida* species need to be incubated for no longer than 72 h, cultures that screen for dermatophytes need to be incubated for only 8 days, and cultures screening for all pathogens should be held for 4 weeks. With an increased immunocompromised population and aggressive use of immunosuppressive therapy, fungal cultures have to detect a wide range of organisms. As a result, incubation of fungal cultures for 4 weeks is a common practice in most clinical microbiology laboratories. The extended incubation time not only delays reporting of negative culture results but also increases the chance of recovering a contaminant. Only 7 samples grew fungal species after 14 days of incubation. In all 7 patients, the organisms recovered by culture were determined to be clinically irrelevant by treating physicians. Among the 7 isolates recovered after 14 days, 3 of them were *Penicillium* species. Although *Penicillium* species were the third-most commonly isolated fungal species in our study, the clinical significance of most of these cultures was questionable. Our study showed that on average, 30% of culture-positive samples had a positive fungal smear. Compared to the smear-positive rate of 44% in samples positive for *Scedosporium* species by culture, only 1.2% of samples that grew *Penicillium* species were smear-positive, which raises the question of the clinical relevance of the species recovered during extended incubation.

A previous report examining the incubation time for recovering *H. capsulatum* in its endemic area reported that up to 4 weeks of incubation was required to recover the organism [11]. The samples analyzed in the study included samples from patients who had received anti-fungal treatments before sample collection. Delayed recovered of the dimorphic fungal species was not noted in our patients. In our study, dimorphic fungal species were recovered in 99 samples. The recovered isolates included 72 *Blastomyces dermatitidis*, 15 *Coccidioides immitis*, 9 *H. capsulatum*, and 2 *Sporotrichum schenckii*. Of these samples, 40.4% were found to be culture-positive less than 1 week, 22.2% were positive between 8-10 days, and 37.4% were positive between 11-14 days of incubation. All the samples were collected before initiation of treatment. Our results indicate that fungal treatment can significantly delay the development of a positive fungal culture.

We have shown that clinically significant pathogens were detected in culture-negative samples from 16 patients by ITS PCR. Direct microscopic examination and culture are the traditional approaches for laboratory diagnosis of fungal infections. In clinical microbiology laboratories, specimens submitted for fungal detection first receive staining and microscopic evaluation, followed by culture. The diagnostic sensitivity of this protocol is low. For example, two studies have reported that the detection of *Aspergillus* species among transplant recipients with clinically confirmed invasive aspergillosis was only 25–50% [5, 16]. Culture-independent laboratory tests based on detection of antibodies, antigens, or metabolites have been developed, but their application for diagnosis of invasive fungal infections suffers from lack of specificity, sensitivity, or ability to provide species-level information [12]. ITS PCR followed by sequencing has become the culture-independent method of choice due to its advantage of allowing for improved sensitivity and rapid identification of fungi in clinical specimens [12]. A recent study evaluating the performance of targeted fungal sequencing for culture-independent diagnosis of invasive fungal disease reported that the method had 96.6% sensitivity and 98.2% specificity for detection of pathogens in specimens with known diagnosis and 71.3% diagnostic yield in patients with proven invasive fungal infections based on the European Organization for Research and Treatment of Cancer/Invasive Fungal Infections [17]. In our study, the ITS PCR positive rate was much lower, only 0.2%. Two main factors may have contributed to the low positive rate. First, we included all samples that had been tested with ITS PCR regardless of the clinical suspicion of fungal infections. Because of the reported high sensitivity of ITS PCR for detection of fungi in clinical samples, the test was often used by clinicians as a rule-out test. The number of patients that underwent testing who had true clinical suspicion of fungal infections is unknown. Second, we did not have a minimum requirement for sample size. As ITS PCR was used as the last resort in clinical practice, the sample size for PCR was scant.

Unlike studies examining the fungal culture time published before 2013, our study reviewed data collected in recent years, reflecting the laboratory practice in response to the increased incidence of invasive fungal disease as a result of the expansion of immunocompromised populations. In addition, the utility of ITS PCR as a last resort to assist the diagnosis of fungal infections was reviewed. Our study concluded that extending culture incubation beyond 2 weeks did not generate clinically relevant results. When culture failed to make a laboratory diagnosis, ITS PCR produced clinically significant results.

## Data Availability

The datasets are available by request to the corresponding author.

## Abbreviation

ITS: Internal transcribed spacer
